# Potent *in vitro* and *in vivo* effects of polyclonal anti-human-myeloma globulins

**DOI:** 10.18632/oncotarget.11489

**Published:** 2016-08-22

**Authors:** Aneta Schieferdecker, Ofer Shoshani, Benedikt Westner, Dov Zipori, Boris Fehse, Nicolaus Kröger, Francis Ayuk

**Affiliations:** ^1^ Department of Stem Cell Transplantation, University Medical Center Hamburg-Eppendorf, Hamburg, Germany; ^2^ Current Affiliation: Department of Oncology and Hematology with Section Pneumology, Hubertus Wald Tumorzentrum/ UCCH, University Medical Center Hamburg-Eppendorf, Hamburg, Germany; ^3^ Department of Molecular Cell Biology, Weizmann Institute of Science, Rehovot, Israel; ^4^ Current Affiliation: San Diego Branch, Ludwig Institute for Cancer Research, La Jolla, CA, USA; ^5^ Neovii (formerly Fresenius) Biotech GmbH, Gräfelfing, Germany; ^6^ Current Affiliation: Acino AG, Miesbach, Germany

**Keywords:** myeloma, anti-human-myeloma globulins, AMG, polyclonal antibodies, ATG

## Abstract

**Introduction:**

Multiple myeloma is still incurable in most cases. Polyclonal anti T lymphocyte globulins (ATG) have been reported to kill human myeloma cells in vitro and in mouse models.

**Methods:**

Anti-human-myeloma globulins (AMG) were produced by immunizing rabbits with human myeloma cell lines RPMI-8226 (AMG-8226) or KMS-12-BM (AMG-12-BM). Cytotoxicity of the polyclonal antibodies was analyzed in vitro and in a xenograft NOD-SCID mouse model.

**Results:**

Both AMG had stronger cytotoxicity against myeloma cells compared to ATG. In primary T cells, AMG-8226 showed greater complement-dependent cytotoxicity (CDC) than ATG, whereas complement-independent cytotoxicity did not differ. Effects on non-hematopoietic cell lines were also similar. Competitive blocking assays revealed fourfold more antibodies against CD38 in AMG-8226 compared to ATG. Low concentrations of AMG-8226 and ATG increased ADCC. At higher concentrations, ATG inhibited ADCC more potently than AMG-8226. Combinations of ATG and AMG-8226 with melphalan or bortezomib showed additive to synergistic cytotoxicity on myeloma cells. The cytotoxic effects of AMG and ATG were confirmed in the xenograft NOD-SCID mouse model.

**Conclusion:**

Our data show more potent antimyeloma effects of AMG compared to ATG. These results lay the ground for the development of polyclonal antibodies for the treatment of multiple myeloma.

## INTRODUCTION

The introduction of novel agents like thalidomide, bortezomib and lenalidomide has led to significant improvement of progression free and overall survival of patients with multiple myeloma. However, most patients suffer disease relapse and progression, rendering myeloma incurable in most cases [1].

Over the past years various monoclonal antibodies like Daratumumab (targeting CD38), Elotuzumab (targeting CS1), Siltuximab (binding IL6), Lorvotuzumab (targeting CD56), nBT062 (targeting CD138) and Dacetuzumab (targeting CD40) have shown preclinical and clinical efficacy [2, 3]. Daratumumab and elotuzumab have recently been approved [3–10]. Furthermore, other immunotherapeutic options such as chimeric antigen receptor T-cells [11–13] or bispecific T-cell engagers [14, 15] are under way.

Effects of these “one-antigen-based” therapeutic strategies may be limited due to the very heterogeneous phenotype of myeloma cells [16, 17] and potential escape mechanism of tumor cells. Combination of monoclonal antibodies or polyclonal antibodies may help increase efficacy, avoid and overcome resistance.

Preclinical data have shown that polyclonal anti T lymphocyte or antithymocyte globulins (ATG) can effectively kill myeloma cells in vitro and in vivo [18–22]. Patients undergoing allogeneic hematopoietic stem cell transplantation (HSCT) for myeloma have been reported to have a better response rate despite lower incidence of graft-versus host-disease, if they received ATG as part of conditioning [23, 24]. Since use of ATG is associated with several potentially dangerous side effects, its application in the treatment of multiple myeloma is limited to the setting of allogeneic transplantation.

ATG is derived by immunization of rabbits or horses with human thymocytes or T-cell lines [25]. Thymocytes and T cells are thus the primary target of ATG while the effects on plasma cells are due to antibodies against antigens that are expressed on both plasma cells and T cells or thymocytes. We hypothesized that anti-human-myeloma globulins (AMG), produced by immunization of rabbits with human myeloma cell lines would have increased antimyeloma cytotoxicity without increase in off-target cytotoxicity.

## RESULTS

### Complement-dependent and complement-independent cytotoxicity of ATG and AMGs in myeloma cell lines

**Figure 1:**
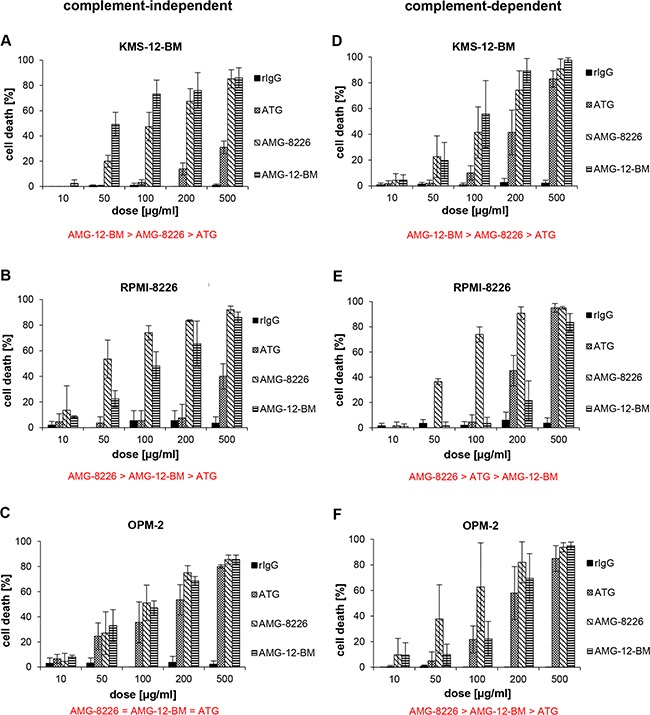
Cytotoxic effect of polyclonals in myeloma cell lines Antithymocyte globulin (ATG) and antimyeloma globulins AMG-8226 and AMG-12-BM induce complement-independent (left column, **A-C**) and complement-dependent cytotoxicity (right column, **D-F**) in myeloma cell lines (KMS-12-BM, RPMI-8226, OPM-2). Rabbit-IgG (rIgG) served as control. Results of statistical analysis (in red): (>) indicates significant superiority with p < 0.05 and (=) indicates equality.

Extensive results of cytotoxicity of the antibodies in human myeloma cell lines are summarized in Figure 1. ATG, AMG-8226 and AMG-12-BM all showed dose-dependent cytotoxicity against all 3 myeloma cell lines tested.

In KMS-12-BM cells, complement-independent cytotoxicity of AMG-12-BM was expectedly higher than for AMG-8226 (p = 0.0001, n = 25) but also higher for AMG-8226 compared to ATG (p = 0.0001, n = 25).

In RPMI-8226 cells, complement-independent cytotoxicity was expectedly higher for AMG-8226 compared to AMG-12-BM (p = 0.0166, n = 10) but also higher for AMG-12-BM compared to ATG (p = 0.008, n = 10).

In OPM-2 cells, complement-independent cytotoxicity of ATG, AMG-8226 and AMG-12-BM did not differ significantly.

Complement-dependent cytotoxicity (CDC) in KMS-12-BM cells was expectedly higher for AMG-12-BM compared to AMG-8226 (p = 0.01, n = 20) but also higher for AMG-8226 compared to ATG (p = 0.0003 n = 20). In RPMI-8226 cells, CDC was expectedly higher for AMG-8226 compared to AMG-12-BM (p = 0.0003, n = 20). Surprisingly, CDC was also higher for ATG compared to AMG-12-BM (p = 0.01, n = 20), although the difference was relatively small and at a low significance level.

In OPM-2 cells CDC was stronger for AMG-8226 compared to AMG-12-BM (p = 0.0045, n = 20) and stronger for AMG-12-BM compared to ATG (p = 0.0005, n = 20).

### Cytotoxic effects of AMG-8226 compared to ATG in primary T cells

**Figure 2:**
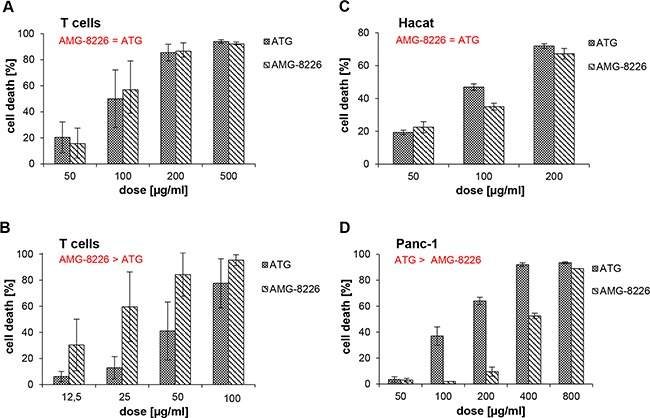
Cytotoxic effect of polyclonals in primary T cells and non-haematopoetic cells In primary T cells, antimyeloma globulin AMG-8226 and antithymocyte globulin ATG induce complement-independent **(A)** and complement-dependent cytotoxicity (CDC) **(B)**, whereby AMG show a significantly stronger CDC compared to ATG (p = 0.0001, n = 20). In Hacat and Panc-1 cell lines (**C** and **D**) AMG does not show higher CDC. Results of statistical analysis (in red): (>) indicates significant superiority with p < 0.05 and (=) indicates equality.

In all, AMG-8226 showed more potent cytotoxicity against myeloma cells compared to AMG-12-BM and was therefore used for further in vitro investigation of off-target effects. T cells were isolated and incubated with ATG or AMG-8226 as described in methods section at doses ranging from 50 to 500 μg/ml without complement and at doses ranging from 12,5 to 100 μg/ml with complement. Complement-independent cytotoxicity did not differ between ATG and AMG-8226 (p = 0.4, n = 20, Figure 2A). AMG-8226 however, showed stronger CDC compared to ATG (p = 0.0001, n = 20, Figure 2B).

### Cytotoxicity of AMG-8226 compared to ATG in non-hematopoetic cell lines

There was no significant difference in cytotoxicity of ATG and AMG-8226 in Hacat cells (p = 0.3, n = 6; Figure 2C). In Panc-1 cells, ATG showed stronger CDC compared to AMG-8226 (p = 0.007; n = 10; Figure 2D).

### Combination effects

**Figure 3:**
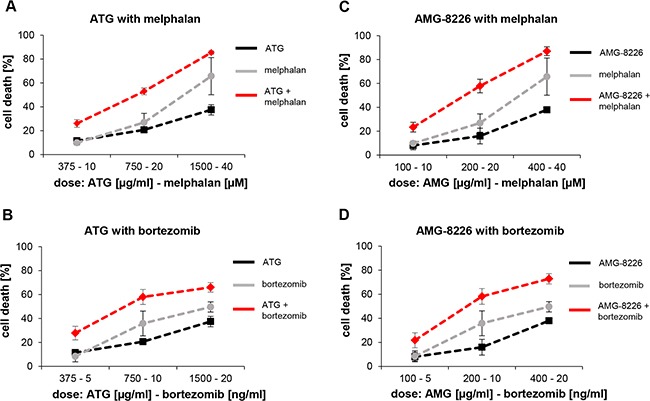
Combination effects of ATG and AMG-8226 with melphalan or bortezomib show additive or synergistic cytotoxic effects KMS-12-BM cells were incubated with ATG or AMG-8226 in combination with melphalan or bortezomib at maximal concentration of IC50 for 24 hours. Cytotoxic effects were measured by flow cytometry. Combination effects were analyzed using CalcuSyn.

Combination of ATG with melphalan or bortezomib revealed additive or synergistic cytotoxic effects on KMS-12-BM cells; combination effects ranged from 0.69 to 0.95 and 0.69 to 1.10 for melphalan and bortezomib respectively (Figures 3A-3B, Supplementary Table S1). Combination of AMG-8226 with melphalan or bortezomib showed similar effects (CI 0.70 to 0.98 and 0.73 to 1.01 respectively; Figures 3C-3D), Supplementary Table S1).

### AMG-8226 contains more anti-CD38 antibodies compared to ATG

CD38 expressing KMS-12-BM cells were incubated with ATG or AMG-8226 prior to staining with anti-CD38 monoclonal antibody as described in methods section.

**Figure 4:**
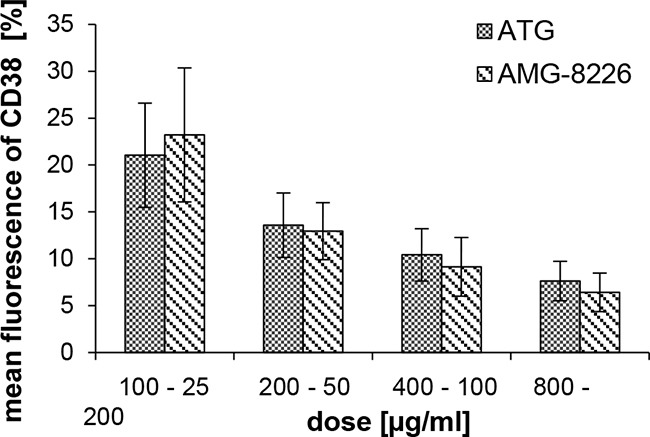
Binding capacity of CD38 CD38 expressing KMS-12-BM cells were pre-incubated with ATG or AMG-8226 prior to staining with anti-CD38 monoclonal antibody. Shown is the dose-dependent reduction in mean fluorescence. The respective dose of ATG was 4 fourfold that of AMG-8226.

Pre-incubation with both AMG-8226 and ATG showed a dose-dependent reduction in mean fluorescence. Equal mean fluorescence was seen, when the concentration of ATG was 4 times that of AMG-8226; this indicates a fourfold higher concentration of anti-CD38 antibodies in AMG-8226 compared to ATG (Figure 4).

### Comparison of ADCC-mediation by ATG and AMG-8226 in chromium release assay

**Figure 5:**
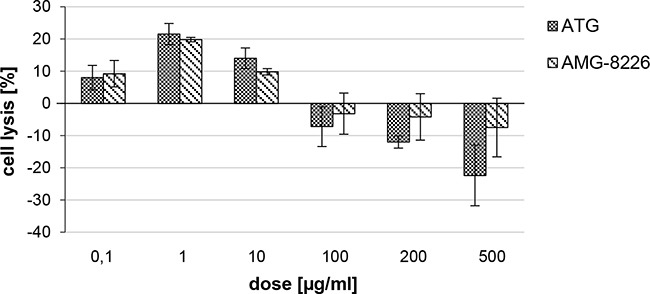
Antibody dependent cellular cytotoxity (ADCC) of ATG and AMG-8226 Cell lysis was measured by chromium release of chromium-51 loaded RPMI-8226 cells after incubation with primary NK cells and ATG or AMG-8226. Cytotoxicity of NK cells alone was set as zero (0).

At low concentrations (0.1-10 μg/ml), both ATG and AMG-8226 increased ADCC (21% and 20% at 1 μg/ml). At higher concentrations (100-500 μg/ml) both polyclonals inhibited ADCC, in which case stronger inhibition was seen with ATG compared to AMG (22% vs. 8% at 500 μg/ml; Figure 5).

### AMG-8226 and AMG-12-BM significantly inhibit myeloma growth in a xenograft NOD-SCID mouse model

**Figure 6:**
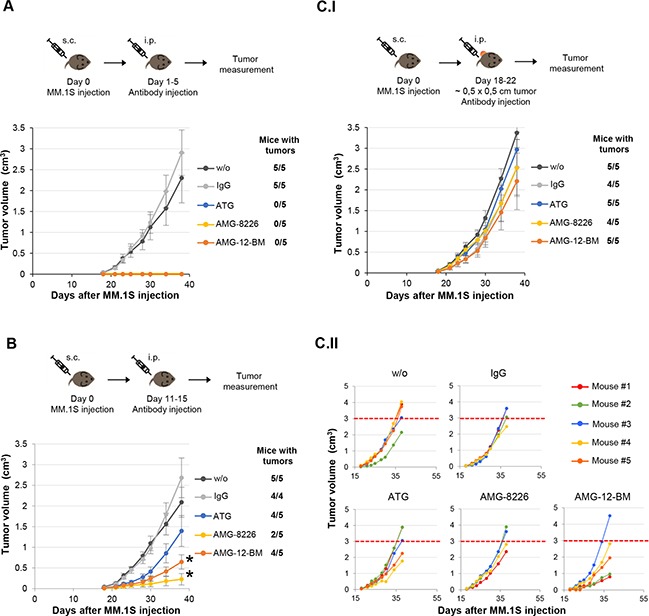
Increased effect of anti-myeloma (AMG) compared to anti-T lymphocyte globulins (ATG) in vivo MM.1S were injected on day 0, and globulin treatment was initiated on days 1 (A), 11 (B), or upon appearance of measurable tumor on day 18 (C), for a duration of 5 days. The mice were followed for a duration of 38 days, and tumor incidence and size were documented every other day. **A.** Treatment starting on day 1 completely prevented any tumor formation, compared to non-treated or IgG treated mice. **B.** Treatment starting on day 11 significantly inhibited tumor growth in both ATG and AMG treatments (statistics are shown in Supplementary Table S2). However, the effect of AMG treatment was higher and more significant. **(C.I)** Treatment starting in mice with tumors of ∼0.5×0.5cm did not show a significant difference over controls. However, when examining individual mice **(C.II)**, AMG-12B-M treatment presents a clear difference in 3 of the mice, in which tumor size was much smaller then the average ∼3cm^3^ tumor size on day 38 (dashed red line).

Early treatment with either ATG or AMG starting on day 1 completely prevented the formation of myeloma tumor in the mice (Figure 6A). When treating at a later time point, (starting on day 11), there was a significant inhibition of tumor growth in both ATG and AMG treated mice. Importantly, treatment with either AMG resulted in a stronger growth inhibition compared to ATG (Figure 6B, and Supplementary Table S2). Finally, treatment after appearance of tumor (∼0.5×0.5cm^3^) did not show a statistically significant effect. However, in 3/5 of AMG-12-BM treated mice a strong inhibition of tumor growth was observed (Figure 6C.II).

## DISCUSSION

Despite significant developments over the past decade, treatment for multiple myeloma is still unsatisfactory with most patients finally succumbing to the disease or treatment related complications.

We hypothesized that using plasma cell lines instead of the Jurkat T cell line to immunize rabbits will produce polyclonal antibodies (AMG) with similar or less off-target effects and more potent antimyeloma cytotoxicity compared to ATG.

Our results show that AMG-8226 has stronger in vitro and in vivo cytotoxicity against myeloma compared to ATG. This is probably due to a higher content of antibodies targeting myeloma cells, as shown for anti-CD38. Cytotoxicity of AMG-8226 on T cells was at least as potent as seen with ATG. Application of AMG is expected to cause profound T cell depletion; therefore, its clinical use can best be envisioned in the context of autologous or allogeneic stem cell transplantation. Effects on non-hematopoietic cells were similar for ATG and AMG. This indicates that the more potent cytotoxicity of AMG is not due to a higher content of antibodies targeting broadly expressed (e.g. HLA) antigens. However, stronger off-target cytotoxicity of AMG in other tissues not examined in our study cannot be ruled out.

In the experiments combining antibodies and NK cells, incubation for 4 hours without complement ensured that neither apoptosis nor CDC occured. In this setting, low concentrations of ATG and AMG both enhanced ADCC. Higher concentrations of both polyclonals inhibited ADCC; however, the inhibitory effect of AMG was much weaker. This finding may be relevant for the in vivo efficacy. AMG-12-BM elicited more complement-independent cytotoxicity than CDC independent of the cell line tested, whereas this varied for ATG and AMG-8226. The differential contribution of complement-independent cytotoxicity, ADCC and CDC to total cytotoxicity may therefore depend on the polyclonal antibody and the target cells involved.

The differences in efficacy seen between AMG-8226 and AMG-12-BM imply that there is still room for further optimization through better choice of the myeloma cell line used for immunization. Moreover, such a cell line could be further genetically modified to increase the expression of myeloma-associated antigens such as CD38 or CD138. Genome editing with engineered nucleases could also be used to decrease the expression of shared or ubiquitously expressed antigens such as HLA molecules, thereby reducing cytotoxic effects on other cells and tissues. AMG produced using such an optimized cell line will be expected to elicit even more potent antimyeloma cytotoxicity but with less off-target side effects.

In conclusion, our study therefore lays the groundwork for further development of polyclonal anti-human-myeloma globulins, which may hold the potential to improve the fate of multiple myeloma patients.

## MATERIALS AND METHODS

### Cell lines

RPMI-8226 (ACC 402), KMS-12-BM (ACC 551) and OPM-2 (ACC 50) were purchased from DSMZ (Braunschweig, Germany). MM.1S (CRL-2974) and PANC-1 (CRL-1469) were obtained from ATCC (Manassas, VA, USA). HaCat were purchased from CLS (catalog no. 300493, Eppelheim, Germany). All cell lines were passaged for less than 6 months after receipt and cultured as recommended.

### Primary T cells and NK cells

Buffy coats were obtained from healthy donors after informed consent. Cell enrichment was performed using RosetteSep^®^ human T cell or NK cell enrichment cocktail (StemCell Technologies Inc, Köln, Germany) according to manufacturer instructions.

### Preparation and purification of anti-human-myeloma globulins

Five rabbits were immunized for each AMG (AMG-12-BM, AMG-8226). Anti-human-myeloma globulins (AMG) were prepared und purified out of whole blood of immunized rabbits. Rabbit sera were obtained by centrifugation of clotted whole blood, heat inactivated and pooled. To decrease cross reactivity, pooled sera were incubated with pasteurized human placenta homogenate, followed by two incubations with human erythrocytes out of erythrocyte concentrate from the German Red Cross. After each incubation step sera were centrifuged.

The absorbed sera were precipitated with ammonium sulfate solution, ultra-centrifuged, decanted and washed with ammonium sulfate solution. Precipitates were dissolved in tris (hydroxymethyl)-aminomethane (TRIS) buffer solution. Ammonium sulfate was removed by dialysis against TRIS buffer, followed by fractionized preparative anion exchange chromatography. Fractions with IgG purity >97% were pooled and pH decreased. Salt residues were removed by dialysis against 10 mM sodiumphosphate buffer pH 3.7. IgG concentration was then adjusted to 18 – 20 mg IgG/mL by ultrafiltration and the purified AMG stored at -80°C.

### Antibodies and reagents

ATG-Fresenius was obtained from Neovii (formerly Fresenius) Biotech GmbH (Gräfelfing, Germany). Rabbit IgG was purchased from Becton Dickinson BD (REF 731642, Heidelberg, Germany).

Bortezomib (Velcade^®^, Ch-B: 7HZT200) was purchased from Janssen-Cilag (High Wycombe, Buckinghamshire, UK) and melphalan (Alkeran^®^, Fluka-63648) from BioChemika Sigma-Aldrich (St. Louis, USA). Active human serum as source of complement was produced from peripheral blood of five healthy donors (after informed consent), pooled and stored at -80°C.

### Viability assays

For complement-independent cell death, cells at a final concentration of 1 × 10^6^ cells / ml were cultured with rabbit IgG, ATG, AMG-8226 or AMG-12-BM as indicated in results and were incubated for 24 hours at 37°C and 5% CO_2_.

For complement-mediated cell lysis, cells were cultured at final concentrations of 1 × 10^6^ cells / ml with IgG, ATG, AMG-8226, or AMG-12-BM as indicated in the results section. After adding 50% complement (active human serum), cells were incubated for 45 minutes at 37°C and 5% CO_2_.

For combination effects, cells were cultured at final concentrations of 1 × 10^6^ cells / ml with ATG or AMG-8226 and melphalan or bortezomib for 24 hours at 37°C and 5% CO_2_. In order to be able to detect combination effects, drug concentrations were chosen below IC50. ATG, bortezomib and melphalan were used in clinically relevant concentrations [26–28].

After the respective incubation periods cells were washed with PBS, 4 μl 7-AAD (catalog no. 559925, BD Biosciences, Heidelberg, Germany) were added, and cells were incubated for 5 minutes at room temperature prior to detection of viable cells by flow cytometry using BD FACS Canto II and BD FACSDiva software (BD Bioscience). Cell viability was expressed as percentage of control: (x − control) / (100 − control) × 100. For statistical analysis Wilcoxon rank sum test was applied. Combination effects were analyzed using CalcuSyn^®^ (Version 2.1, Biosoft, Cambridge, UK) based on the method of T.C. Chou and P. Talalay [29].

### Competitive blocking assays

1×10^6^ CD38-expressing myeloma cells (KMS-12-BM) were incubated with 10% AB-Serum (Anti-AB, BioClone^®^ Ortho-Clinical Diagnostics Inc, Neckargemünd, Germany) for 30 minutes at room temperature to reduce non-specific binding. After washing with PBS, cells were incubated with ATG or AMG-8226 for 20 minutes at 4°C and thereafter washed with PBS prior to staining with 10 μl anti-human CD38-PE (BD Bioscience, REF 345806, clone HB7) for 20 minutes at 4°C and flow cytometry.

### Chromium-release assay

In order to measure antibody-dependent cellular cytotoxicity (ADCC) of ATG and AMG-8226, target cells (myeloma cell line RPMI-8226) were incubated with Chromium-51 (Hartmann Analytic GmbH, Braunschweig, Germany) at a concentration of 100μCi ^51^Cr / 1×10^6^ cells for 1.5 hour at 37°C. After three washing steps primary NK cells were added at effector-target ratio 5:1 with ATG or AMG-8226 in doses ranging from 0,1 to 500 μg/ml. After incubation for 4 hours at 37°C Chromium-51 activity in the supernatant was measured using a γ-counter (1470 WallacWizard, PerkinElmer, Wattham, USA). Cell lysis was expressed in Chromium-51 release as a percentage of control (NK cells and target cells alone): (x − control)/ (100 − control) × 100.

### In vivo mice experiments

Mouse experiments were performed in Prof. Zipori's group at the Weizmann Institute. The Weizmann Institutional Animal Care and Use Committee approved all animal experiments (approval #: 04711005-2). MM.1S cells were cultured in RPMI medium with 10% FCS. Cells were harvested and washed twice with PBS prior to injection. Eight to ten week old female NOD-SCID mice were subcutaneously injected 1×10^6^ MM1S myeloma cells over their shaved right flank. Six mice each were then randomly assigned to one of 5 groups that were either untreated (control), or treated with rabbit IgG, ATG, AMG-8226 or AMG-12-BM administered intraperitoneally at 5mg/kg/day. Tumors were measured using a caliper, and volumes were calculated by length × (breadth)^2^/2. Mice were monitored till day 38 or killed when tumor volume exceeded 2.5 cm^3^. One-way ANOVA and Tukey HSD analysis were used to determine significance. A value of p<0.05 was considered statistically significant.

## SUPPLEMENTARY MATERIAL TABLES




