# Supplementary material for: Potent in vitro and in vivo effects of polyclonal anti-human-myeloma globulins
Source: Oncotarget. 2016 Aug 22;7(41):67061–70. doi: 10.18632/oncotarget.11489 (PMC5341857; doi:10.18632/oncotarget.11489)
Supplement: Supplementary file 1 [file oncotarget-07-67061-s001.pdf]

Potent in vitro and in vivo effects of polyclonal anti-human-myeloma globulins

SUPPLEMENTARY TABLES

Supplementary Table S1: Combination effects (CI) of ATG and AMG-8226 with melphalan or bortezomib analyzed using CalcuSyn (Version 2.1, Biosoft, Cambridge, UK)

Combinationindices (CI) of ATG and melphalan.

ATG (µg/ml)	melphalan (µM)	CI	effect
375	10	0,946	nearly additive
750	20	0,902	nearly additive
1500	40	0,686	synergism

Combinationindices (CI) of AMG-8226 and melphalan.

AMG-8226 (µg/ml)	melphalan (µM)	CI	effect
50	10	0,978	nearly additive
100	20	0,835	moderate synergism
200	40	0,699	synergism

Combinationindices (CI) of ATG and bortezomib.

ATG (µg/ml)	bortezomib (ng/ml)	CI	effect
375	5	0,854	slight synergism
750	10	0,693	synergism
1500	20	1,096	nearly additive

Combinationindices (CI) of AMG-8226 and bortezomib.

AMG-8226 (µg/ml)	bortezomib (ng/ml)	CI	effect
50	5	1,008	nearly additive
100	10	0,725	moderate synergism
200	20	0,959	nearly additive

Data were interpreted in accordance with published CI ranges of Chou [29].

Supplementary Table S2: Significance (p-val) of tumor size difference at interval days calculated using One-way ANOVA Standard weighted analysis, Tukey HSD.

See Supplementary File 1
