# Supplementary material for: Potent in vitro and in vivo effects of polyclonal anti-human-myeloma globulins
Source: Oncotarget. 2016 Aug 22;7(41):67061–70. doi: 10.18632/oncotarget.11489 (PMC5341857; doi:10.18632/oncotarget.11489)
Supplement: Supplementary file 2 [file oncotarget-07-67061-s002.docx]

| **Day 18** |  |  |  |  | **Day 28** |  |  |  |  |
| --- | --- | --- | --- | --- | --- | --- | --- | --- | --- |
|  |  |  |  |  |  |  |  |  |  |
|  | **+ATG** | **+AMG-8226** | **+AMG-12-BM** |  |  | **+ATG** | **+AMG-8226** | **+AMG-12-BM** |  |
| **MM1.S** | <0.05 | <0.05 | <0.05 |  | **MM1.S** | <0.01 | <0.01 | <0.01 |  |
| **+IgG** | n.s. | n.s. | n.s. |  | **+IgG** | <0.01 | <0.01 | <0.01 |  |
|  |  |  |  |  |  |  |  |  |  |
|  |  |  |  |  |  |  |  |  |  |
| **Day 21** |  |  |  |  | **Day 30** |  |  |  |  |
|  |  |  |  |  |  |  |  |  |  |
|  | **+ATG** | **+AMG-8226** | **+AMG-12-BM** |  |  | **+ATG** | **+AMG-8226** | **+AMG-12-BM** |  |
| **MM1.S** | n.s. | <0.05 | n.s. |  | **MM1.S** | <0.01 | <0.01 | <0.01 |  |
| **+IgG** | n.s. | n.s. | n.s. |  | **+IgG** | <0.05 | <0.01 | <0.01 |  |
|  |  |  |  |  |  |  |  |  |  |
|  |  |  |  |  |  |  |  |  |  |
| **Day 23** |  |  |  |  | **Day 34** |  |  |  |  |
|  |  |  |  |  |  |  |  |  |  |
|  | **+ATG** | **+AMG-8226** | **+AMG-12-BM** |  |  | **+ATG** | **+AMG-8226** | **+AMG-12-BM** |  |
| **MM1.S** | <0.05 | <0.01 | <0.01 |  | **MM1.S** | n.s. | <0.01 | <0.01 |  |
| **+IgG** | n.s. | <0.01 | <0.05 |  | **+IgG** | <0.05 | <0.01 | <0.01 |  |
|  |  |  |  |  |  |  |  |  |  |
|  |  |  |  |  |  |  |  |  |  |
| **Day 25** |  |  |  |  | **Day 38** |  |  |  |  |
|  |  |  |  |  |  |  |  |  |  |
|  | **+ATG** | **+AMG-8226** | **+AMG-12-BM** |  |  | **+ATG** | **+AMG-8226** | **+AMG-12-BM** |  |
| **MM1.S** | <0.01 | <0.01 | <0.01 |  | **MM1.S** | n.s. | <0.01 | <0.05 |  |
| **+IgG** | <0.01 | <0.01 | <0.01 |  | **+IgG** | n.s. | <0.01 | <0.01 |  |

**Supplementary Table 2**: Significance (p-val) of tumor size difference at interval days calculated using One-way ANOVA Standard weighted analysis, Tukey HSD.
